# Using cell‐free RNA to identify B‐ and T‐cell clonality for diagnosis and monitoring of B‐ and T‐cell neoplasms

**DOI:** 10.1002/2211-5463.70323

**Published:** 2026-08-13

**Authors:** Adam Albitar, Sally Agersborg, Ahmed Charifa, Pooja Phull, Noa Biran, David Vesole, Harsh Parmar, Andrew Pecora, Andrew Ip, Andre Goy, David Siegel, Maher Albitar

**Affiliations:** ^1^ Genomic Testing Cooperative Lake Forest CA USA; ^2^ John Theurer Cancer Center, Hackensack University Medical Center NJ USA

**Keywords:** B‐cell clonality, cell‐free RNA, liquid biopsy, lymphoid neoplasm, T‐cell clonality. clonotype

## Abstract

Cell‐free RNA (cfRNA) is emerging as a supplemental approach for liquid biopsy (LBx). Given that lymphoid cells and plasma cells express substantial quantities of immunoglobulin (Ig) and T‐cell receptor (TCR) RNA, we investigated the utility of using cfRNA for detecting B‐cell and T‐cell clonality in LBxs. Using next‐generation sequencing (NGS) of cfRNA, we clonotyped Igs and TCRs in LBx samples from patients with B‐cell neoplasm, T‐cell neoplasm, myeloid neoplasm and solid tumors alongside cancer‐free individuals. To establish a clonality cutoff, we clonotyped tissue RNA from patients with confirmed clonal B‐ or T‐cell and from polyclonal. Clonotype‐naïve testing of LBx samples demonstrated B‐cell clonality in 36% of B‐cell neoplasms, 7% of normal, 8% of T‐cell lymphomas, 13% of myeloid neoplasms, and 14% of solid tumors. T‐cell clonality using TCR beta or gamma demonstrated T‐cell clonality in 22% of T‐cell neoplasms, 3% of normal, 5% of myeloid neoplasms, 7% of solid tumors and 3% of B‐cell neoplasms. This confirms that Clonotype‐naïve cfRNA testing in LBx is a reliable approach for detecting B‐ and T‐cell clonality.

## Graphical abstract

Using peripheral blood for determining B‐cell or T‐cell clonality is more reliable when we use cell‐free RNA (cfRNA) because cells release blood significantly more RNA than DNA. Next‐generation sequencing (NGS) of cfRNA allows us to evaluate fragment cfRNA and evaluate clonality reliably without the need for prior determination of the specific dominant clone (clonotype‐agnostic).

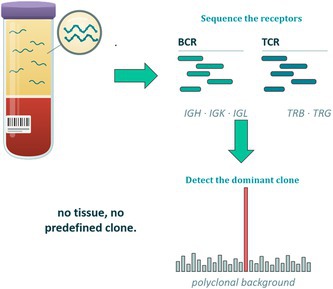

**Abbreviations**
cfDNA: cell‐free DNA
cfRNA: cell‐free RNA
CHIP: clonal hematopoiesis of indeterminate potential
Ig: immunoglobulin
LBx: liquid biopsy
NGS: next‐generation sequencing
TCR: T‐cell receptor

Detection of B‐ and T‐cell clonality in various lymphoid neoplasms is crucial for diagnosis and monitoring of these neoplasms. Heavy and light chain immunoglobulins (Igs) as well as T‐cell receptors (TCRs) alpha (TRA), beta (TRB), and gamma (TRG) are frequently analyzed to determine clonality using either polymerase chain reaction (PCR) or next‐generation sequencing (NGS) [1, 2, 3, 4, 5]. The random process of rearranging Variable (V), Diversity (D—for heavy chains only), and Joining (J) Ig and TCR gene segments produces massively diverse, unique sequences that are used as molecular fingerprints to identify the presence of clonal lymphoproliferations. Typically, clonality testing is performed on cellular DNA using fragment length analysis, amplicon based NGS, and to a lesser degree, capture based NGS. Each of these methodologies comes with its own inherent benefits and limitations though recent studies suggest that using hybrid capture can be more accurate and more reliable for detecting Ig/TCR clonality [4, 6, 7, 8].

Using cell‐free DNA (cfDNA) in tumor‐naïve clonality assessment for lymphomas has been reported but is rarely used in routine practice, instead relying on tissue biopsy. This is due to poor sensitivity in low tumor burden settings and difficulties with sequencing in tumor types with ongoing somatic hypermutation [9, 10, 11].

RNA has been used to profile Ig/TCR repertoire in various immune processes but is rarely used in evaluating clonality in lymphoid neoplasms [12, 13]. There are numerous advantages for using RNA over DNA in evaluating Ig/TCR clonality. For example, each cell typically contains numerous copies of light and heavy Ig or TCR mRNA as compared to one copy of rearranged Ig or TCR DNA. RNA is also intronless, and this reduces the interference of the noncoding sequences in sequence analysis. The intronless sequence is also important for increasing efficiency and for capturing a representative high number of clones when NGS is used.

We have reported that cell‐free RNA (cfRNA) analysis using targeted NGS sequencing as a part of liquid biopsy can be reliable and provides quantitative information that can be used to predict specific clinical conditions [14, 15]. Here, we explore the value of quantifying various Ig/TCR expressed clones as detected using cfRNA and attempt to establish Ig/TCR clonality criteria for the diagnosis and screening for lymphoid neoplasms using LBx. The ability to demonstrate clonality using peripheral blood cfRNA can provide an alternative to invasive tissue and bone marrow sampling and may allow for improved monitoring of minimal residual disease (MRD). Unlike bone marrow or peripheral blood cells, cfRNA and cfDNA reflect the entire body and are therefore not hindered by the sampling bias.

## Materials and methods

### Samples and RNA extraction

We isolated cfRNA and cfDNA from 1596 consecutive peripheral blood (PB) samples submitted for liquid biopsy testing. All PB samples were collected in EDTA and tested within 72 h of collection. The PB samples were submitted for confirming diagnosis and or for monitoring of disease and MRD. These included various types of hematologic neoplasms and solid tumors without any selection. Based on clinical data and testing results of the liquid biopsy, 671 of these samples were classified as negative for a diagnostic cancer (normal), but they included cases with clonal hematopoiesis of indeterminate potential (CHIP) (Table 1). Cancer diagnosis was confirmed as solid tumor in 98 samples, B‐cell neoplasms in 449 samples, T‐cell neoplasms in 51 samples, and myeloid neoplasms in 327 samples. Diagnosis of the presence of a solid tumor or hematologic neoplasm was confirmed by the presence of tumor‐specific mutations. The sensitivity of the assay was in the range of 0.0001 for hot spot mutations or tumor‐informed samples and 0.01 for other mutations. Table 1 shows the specific diagnosis and classification of these cases. The median age of patients was 66 (range from 3 to 99). Tissue RNA was collected from 551 FFPE samples or fresh bone marrow samples with confirmed B‐cell (*n* = 501) or T‐cell (*n* = 50) lymphoma/leukemia and from 188 normal lymphoid tissue or bone marrow samples and other types of tissue with no evidence of B‐ or T‐cell neoplasms or clonality. The tissue samples were consecutive and included fresh bone marrow and tissue samples. All tissue samples were in FFPE (formalin‐fixed paraffin‐embedded). submitted for molecular diagnosis. These included various types of lymphoma, including multiple myeloma. cfRNA was extracted using Apostle MiniMax High Efficiency cfRNA/cfDNA isolation kit. Tissue RNA was extracted from FFPE tissues using an Agencourt FormaPure Total 96‐Prep Kit and automated KingFisher Flex, following the manufacturer's recommendations.

**Table 1:** Characteristic of the tested samples.

Category	Diagnosis	Number
Normal/CHIP	EBV‐positive lymphocytosis	1
	CHIP	478
	Normal	182
	AA	2
	Hodgkin	8
Total normal/CHIP		671
B‐cell lymphoid	Lymphoma	319
	CLL	31
	Mantle	18
	MM	37
	Follicular	9
	Hairy	2
	Wald	12
	DLBCL	11
	Marginal	1
	MDS‐Mantle	1
	ALL	2
	Gammopathy	6
Total B‐cell lymphoid		449
**T‐cell lymphoid**	**T‐cell**	**51**
Myeloid	MPN	71
	VEXAS	13
	MDS	153
	AML	26
	MDS‐AML	15
	CMML	8
	MDS‐CMML	20
	MPN‐CMML	1
	MDS‐MPN	11
	CNL	1
	VEXAS‐MDS	1
	Mastocytosis	1
	CML	5
	CML‐BC	1
Total myeloid		327
Solid tumors	Endometrial	4
	Thyroid	2
	Lung	11
	Renal	7
	Uterine	1
	Pancreas	3
	Cholangio	2
	Liver	2
	GIST‐L	1
	Melanoma	1
	Sarcoma	2
	Head&Neck	7
	Breast	11
	CLL and‐Breast	1
	Prostate	5
	Colorectal	7
	Upper GI	2
	Urothelial	1
	Ovarian	2
	Squamous	1
	Bile duct	1
	Carcinoma	23
	Histiocytic sarcoma	1
Total solid tumors		98
Total		1596

AA, aplastic anemia; ALL, acute lymphoblastic leukemia; CHIP, clonal hematopoiesis of indeterminate potential; CLL, chronic lymphocytic leukemia; CML‐BC, chronic myeloid leukemia‐blast crisis; CMML, chronic myelomonocytic leukemia; CNL, chronic neutrophilic leukemia; DLBCL, diffuse large B‐cell lymphoma; MDS, myelodysplastic syndrome; MM, multiple myeloma; MPN, myeloproliferative neoplasm; VEXAS, Vacuoles, E1 enzyme, X‐linked, Autoinflammatory, somatic syndrome.

The study protocol was approved by the Institutional Review Board (IRB) of the Western Copernicus Group (New England IRB, Aspire IRB, and Midlands IRB) (Number 1‐1476184‐1). The need for informed consent was waived due to the incidental collection and lack of risk. This study was conducted in accordance with the principles of the Declaration of Helsinki and its amendments.

### Targeted RNA and cfRNA next‐generation sequencing

Tissue RNA and cfRNA were processed for NGS using KAPA RNA HyperPrep (Roche, Indianapolis, IN, USA) kit as recommended by the manufacturer. The cfRNA processing was similar to that of tissue with the omission of nucleic acid fragmentation. These steps are described in more detail in a previous paper (Albitar *et al*., [14]). Hybrid capture sequencing targeted approximately 1600 genes that included all immunoglobulin heavy chain (IgH), Kappa (IgK), and Lambda (IgL) genes as well as all T‐cell receptors alpha (TRA), beta (TRB), and gamma (TRG) genes. Sequencing was performed using the Illumina NovaSeq 6000 instrument. Runs with a minimum of 100 million reads and ≥ 25% splice junction reads were accepted for further evaluation.

After sequencing, Dragen 3.8 RNA seq pipeline for fusion calls and salmon v1.4.0 for expression (TPM) were applied. The RNA sequencing was considered contaminated by DNA and the case was excluded from the study if the percent of splice junction reads was < 25%.

For B‐ and T‐cell repertoire analysis, sequences were analyzed using the mixcr software (https://mixcr.com). Sequences were first mapped to the germline database, then assembled to clonotypes. Since our sequencing generates paired‐end data, mixcr merges and is able to overlap mate pairs even if there is only a single overlapping nucleotide. Low quality nucleotides at read edges were trimmed. In repertoire analysis, mixcr corrects the assembled clones and reports a consensus sequence for each cluster.

### Statistical analysis

For determining the cutoff point, receiver operating characteristic (ROC) analysis was used since the goal was to separate binary outcome (positive vs negative). We opted to increase specificity over sensitivity. For comparing cutoff points and using two vs one gene family, we used the Wilcoxon signed‐rank test. For all analysis, confidence intervals were calculated based on the standard error for the difference between two independent proportions using pooled or unpooled standard error estimates to create a 95% confidence interval.

## Results

### Establishing a cutoff for clonotype‐agnostic Ig/TCR clonality

**Fig. 1:**
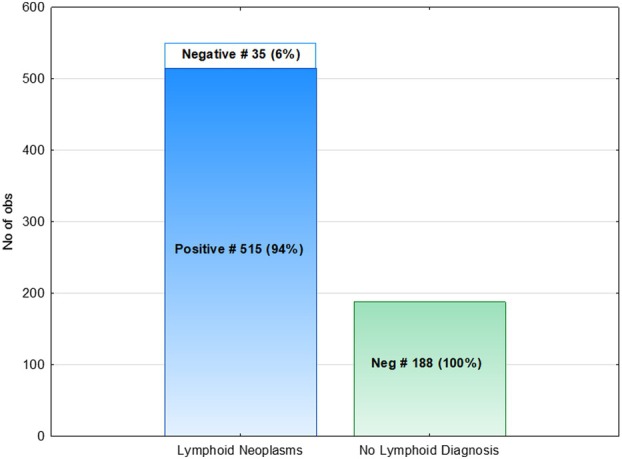
Histogram showing the frequency of clonality of B‐ and T cell as detected in tissue samples using the highest expressed clone: the second highest clone with a ratio of 10 or higher using RNA.

In order to establish a cutoff for clonality in a sample, we evaluated the Ig/TCR repertoire in 739 tissue samples. Of these samples, 188 were confirmed polyclonal for B‐ and T‐cell populations by either flow cytometry or conventional molecular studies as well as lack of mutations associated with lymphoid neoplasm. The rest of the cases included 501 confirmed B‐cell neoplasms and 50 confirmed T‐cell neoplasms. All samples were diagnostic samples with dominant neoplastic tissue. A relative ratio of the highest expressed (TPM) clonotype to the second highest expressed clonotype of 10 showed the highest specificity (100%) with sensitivity of 94% (Fig. 1). Using cutoff of 5 reduced specificity to 63.36% [95% confidence interval (CI): 59.07% to 67.49%]. while using 8 as a cutoff reduced specificity to 80.78% (95% CI: 76.63% to 84.48%). We used the 10 cutoff to assure that clonotype‐agnostic testing is highly specific and to avoid false calling of clonality in clonotype‐agnostic cases. Our goal in using such highly stringent approach is to increase the specificity of detection of MRD in clonoctype‐informed cases. In separate set of 48 clonotype‐informed samples with clonality ratio less than 10, but with residual disease based on DNA mutation profile, all 48 testing samples showed residual disease by mutation profiling (100% sensitivity). In 19 clonotype‐informed samples that were negative by mutation profiling, IgHV profiling showed no evidence of residual disease in 18 of the 19 samples.

### Establishing sensitivity of Ig/TCR clonality detection

To evaluate sensitivity of detecting clonality of Ig or TCR, we compared detection of clonality with detection of mutations. Mutation percentages represent the percentage of DNA in the sample more accurately than RNA since RNA expression may vary dependent on the cell type and level of differentiation. Serial samples collected over a period of time from a patient with B‐cell lymphoma and from a patient with T‐cell lymphoma with specific driver mutations were used. Ig heavy chain and light chain kappa and TRG clonality were compared with variant allele frequency of driver mutations. In the patient with T‐cell lymphoma, we compared TRG clonality with a driving mutation in PHF6 gene using 9 serial samples collected at different time points. In the patient with B‐cell lymphoma, we compared IgH and IgK clonality with BRAF mutation in 6 serial samples collected at different time points. The difference between time points was 3 to 6 months.

**Fig. 2:**
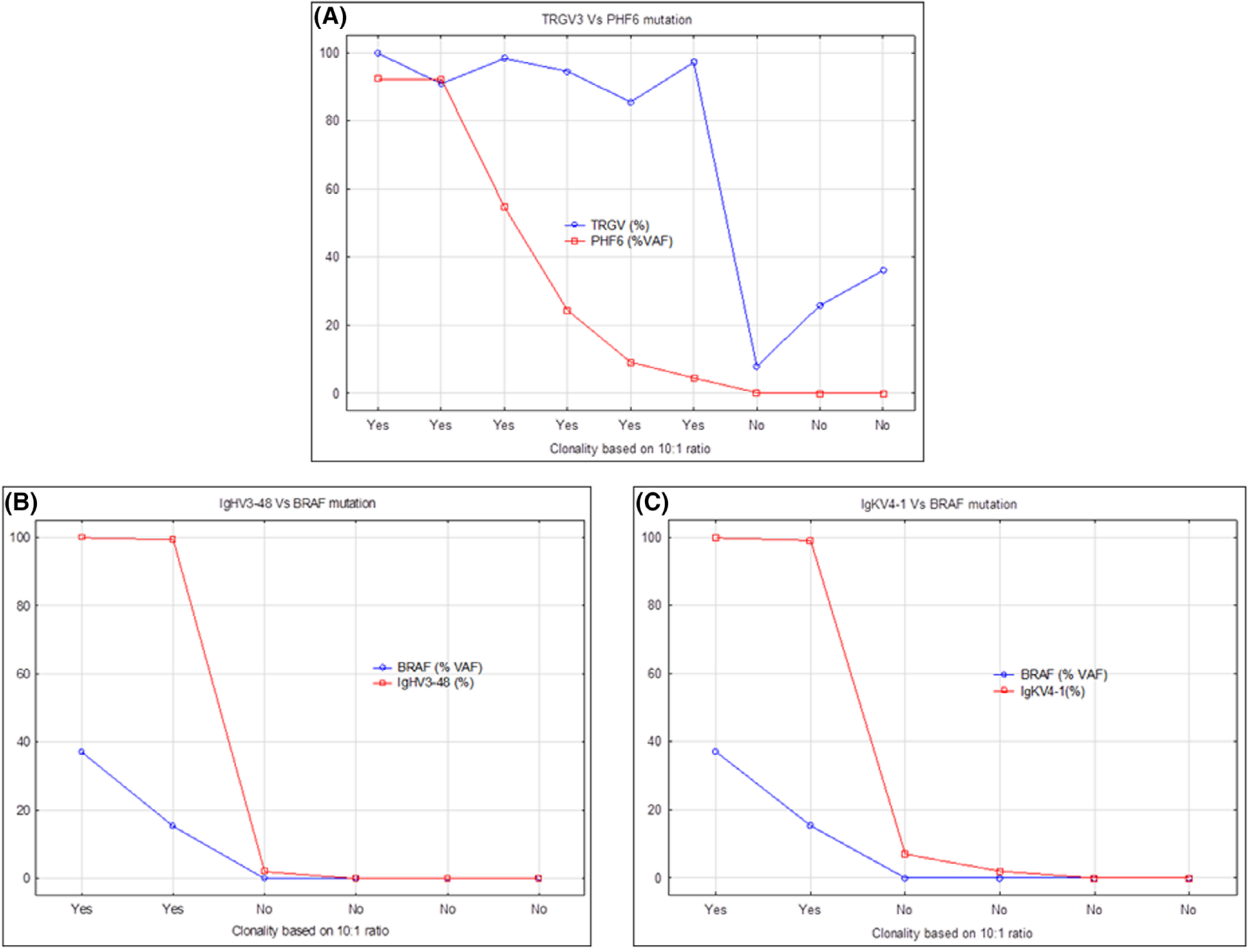
Comparing T‐cell receptor (TCR) and immunoglobulin (Ig) sensitivity with mutation sensitivity: Panel A shows the percentage of T‐cell receptor gamma (TRG) and the variant allele frequency of PHF6 mutation in the same sample. Nine different time points are shown. The *X*‐axis represents different testing time points. For each time point, it is stated if clonality based on 10‐fold was detected (Yes) or not (No). Panel B shows a similar comparison between immunoglobulin heavy chain (IgH) clonality and BRAF mutation. Panel C shows testing of the same samples in B at the same time points but compares light chain Kappa (IgK) with BRAF mutation.

As shown in Fig. 2A, the T‐cell clonality remained detectable using our criteria of 10 : 1 ratio even when the mutation variant allele frequency (VAF) level was at 0.23%. The defined clone (TRGV3) was also detectable in subsequent 3 samples when the PHF6 mutation VAF was not detectable, confirming significantly higher sensitivity of clonotype‐informed testing (Fig. 2A). In the B‐cell lymphoma case, the BRAF mutation was detectable at VAF of 37% and 15%, in which the IGH and IGK clonality was detectable using our 10 : 1 ratio (Fig. 2B,C). However, when the BRAF mutation was not detectable, both IGH and IGK clonality based on 10 : 1 was not detectable, but the defined IGHV3‐48 and IGKV4‐1 clones were still detectable (Fig. 2B,C). The sensitivity of detecting PHF6 or BRAF mutations was determined to be at 1 : 10 000 based on mixing studies. These data suggest that sensitivity of clonality in a clonotype‐agnostic fashion using a ratio of 10 : 1 is around 0.2%, while the sensitivity of clonotype‐informed testing is better than 1 : 10 000.

### B‐cell clonality using cfRNA expression of immunoglobulin heavy and light chain

**Fig. 3:**
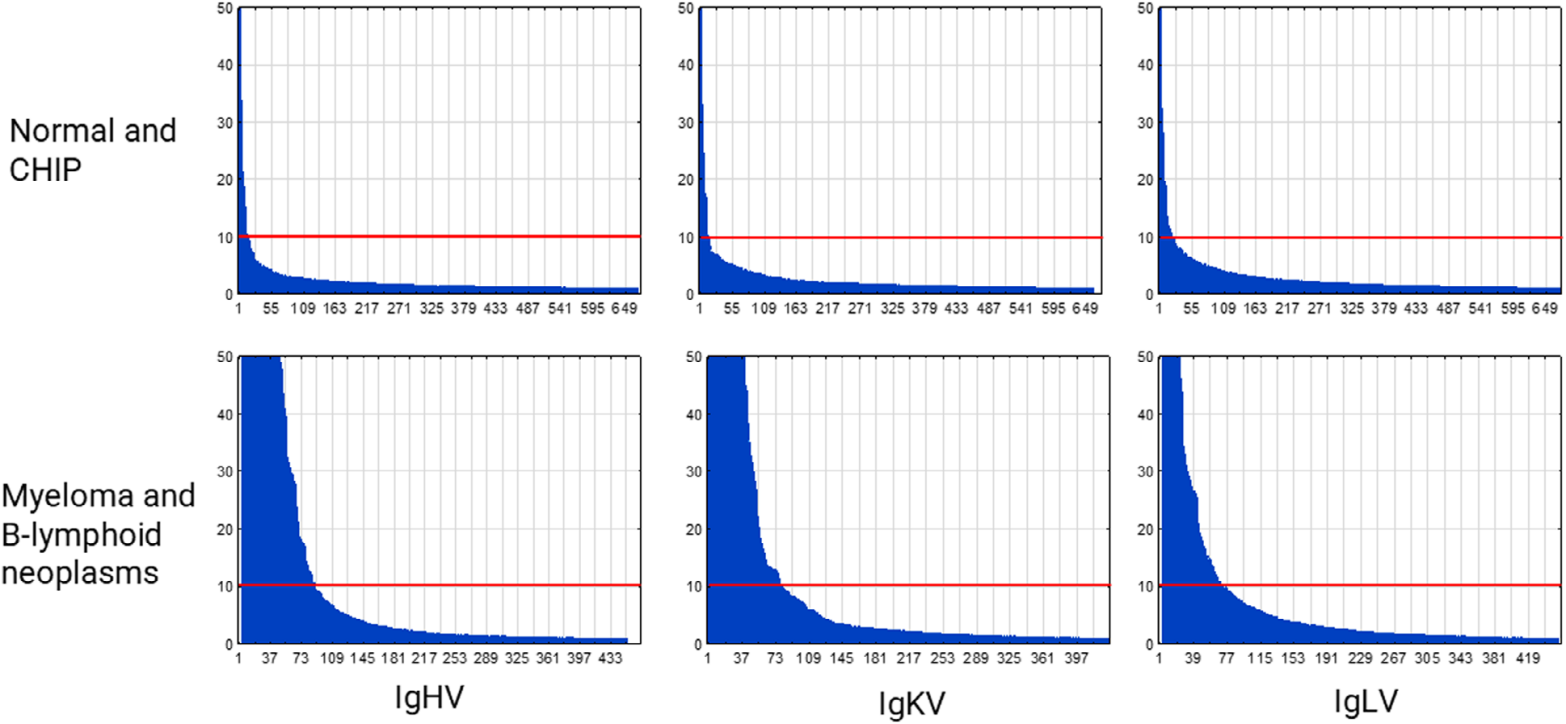
Plots showing the ratios of the highest expressed clone: the second highest clone of heavy and light chains in normal/clonal hematopoiesis of indeterminate potential (CHIP) vs. multiple myeloma and B‐cell neoplasms. Samples are sorted from the highest to lowest. The *X*‐axis represents the number of tested cases. The *Y*‐axis shows the ratios. The red line represents the cutoff point of 10 used for determining positive vs negative.

Testing 449 samples with clinical data and cfDNA evidence of B‐cell lymphoma or multiple myeloma (Table 1) showed 86 of 449 (19%) samples with cfRNA expression of an IgH clonotype that meets the 10‐fold cutoff (Table 2). In contrast, only 16 of the 671 (2%) normal/CHIP cfRNA samples demonstrated an IgH clonotype. Figure 3 shows the ratios in all cases and compares the pattern with normal/CHIP cases. As shown in Table 2, clonality by IgH was detected in 5% of cases with solid tumors, 3% of myeloid neoplasms, and 4% of T‐cell lymphoid neoplasms. Using a less stringent cutoff point would have increased the percentage of clonal cases in both B‐cell cases and normal/CHIP cases. Reducing the cutoff ratio to ≥ 5 increased positivity by 9% in lymphoid cases and by 4% in normal/CHIP cases.

**Table 2:** B‐ and T‐cell clonality of various samples.

	Number	Clonal by IgH	Clonal by IgK	Clonal by IgL	Clonal by any B‐cell receptor	Clonal by TRA^a^	Clonal by TRB	Clonal by TRG	Clonal by any T‐cell receptor	Clonal by TRB or TRG or both
Normal/CHIP	671	2%	2%	3%	7%	17%	1%	2%	20%	3%
B‐cell	449	19%	19%	16%	36%	10%	1%	2%	13%	3%
T‐cell	51	4%	4%	4%	8%	14%	12%	12%	33%	22%^b^
Myeloid	327	3%	5%	7%	13%	13%	2%	4%	18%	5%
Solid	98	5%	8%	6%	14%	16%	2%	7%	22%	7%

^a^ If we exclude the TRAV1‐1 gene family in calculating clonality by TRA, T‐cell clonality was not detected in normal/CHIP, B‐cell neoplasm, or myeloid neoplasm by TRA and detected in T‐cell neoplasms at 4% and in solid tumors at 2%

^b^ If we exclude TRAV1‐1 and use TRA, T‐cell clonality is detected at 25% in cases with T‐cell lymphoid neoplasms.

B‐cell clonality data as detected using the light chains is shown in Table 2 and Fig. 3. A higher percentage of clonality is detected using the IgK expression as compared to IgL in B‐cell lymphoid neoplasms (19% vs 16%). In normal/CHIP cases, 3% were clonal by IgL and 2% were clonal by IgK. Low‐level clonality by light chain is also detected in non‐B‐cell neoplasms (Table 2).

### Improving detection of B‐cell clonality using either heavy or light chains

Assessing clonality by any B‐cell receptor clonotype (heavy chain or light chain) improved detection significantly from 19% to 36% (*P* < 0.0001). Only 3 (0.6%) of cases demonstrated clonality in both IgK and IgL, and 23 (5%) cases showed clonality in IgH only. In normal/CHIP cases, 7% of cases showed clonality by either heavy chain or light chain or both. Similarly, detection of B‐cell clonality increased slightly in myeloid, T‐cell neoplasms, and solid tumors (13, 14, and 8%, respectively) when assessing clonality by any B‐cell receptor (Table 2).

### T‐cell clonality using cfRNA expression of T‐cell receptors

**Fig. 4:**
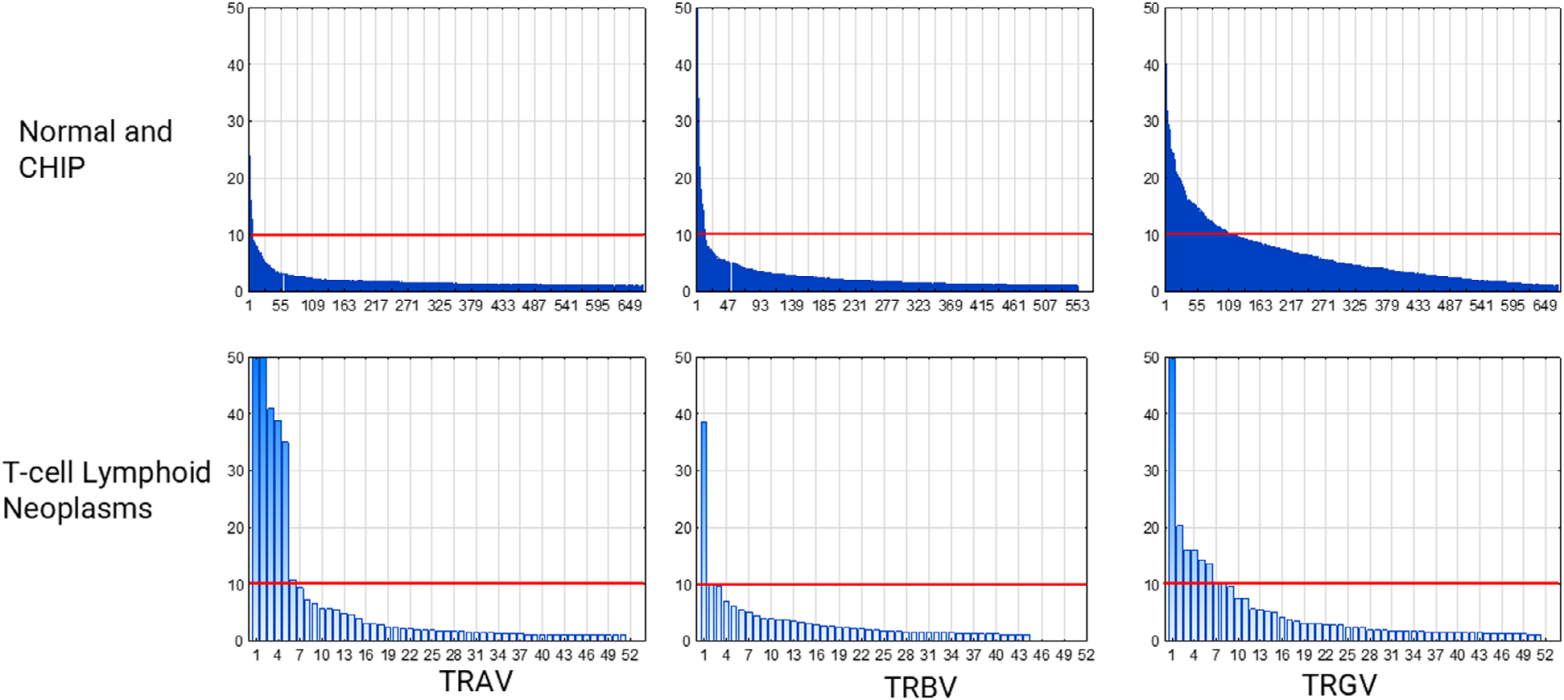
Plots showing the ratios of the highest expressed clone: the second highest clone of T‐cell receptors in normal/clonal hematopoiesis of indeterminate potential (CHIP) vs. T‐cell neoplasms. Samples are sorted from the highest to lowest. The *X*‐axis represents the number of tested cases. The *Y*‐axis shows the ratios. The red line represents the cutoff point of 10 used for determining positive vs negative.

Using the same clonality cutoff ratio of 10‐fold, TRA showed clonality at high percentage in all types of neoplasms (Table 2 and Fig. 4). A TRAV1‐1 clone was detected at high level in the majority of these cases. If we exclude cases with a TRAV1‐1 clonotype, only two cases with T‐cell neoplasm (5%) and two cases with solid tumors (2%) show clonality by TRA. None of the normal/CHIP, B‐cell neoplasms, or myeloid neoplasms showed clonality using TRA if we exclude the TRAV1‐1 gene family. This reflects the limited diversity in the T‐cell receptor alpha repertoire. T‐receptor beta and gamma were more specific in detected clonality. T‐cell clonality using TRB was at 1% in normal/CHIP cases and B‐lymphoid cases and at 12% in T‐cell lymphoid neoplasms. In solid tumors and myeloid tumors, clonality was detected at 2% using our 10‐fold cutoff (Table 2 and Fig. 4). Similarly, clonality using TRG was specific and detected in normal/CHIP and B‐lymphoid cases at 2%, T‐lymphoid cases at 5%, solid tumors at 7%, and myeloid tumors at 4%.

### Improving detection of T‐cell clonality using TRB or TRG

Using either TRB or TRG, 22% of T‐cell cases showed clonality, while only 3% of normal/CHIP cases, 5% of myeloid cases, and 7% of solid tumors showed clonality.

Including TRA increased the clonality in T‐lymphoma cases to 33%, but specificity was low because clonality was also detected in normal/CHIP cases at 20% and in solid tumors at 22% (Table 2). However, if we exclude TRAV1‐1 and use other TRA families to improve specificity, T‐cell clonality is detected at 25% in cases with T‐cell lymphoid neoplasms.

## Discussion

Detecting Ig/TCR clonality is important for the diagnosis, monitoring, and therapeutic planning in lymphoid neoplasms and in multiple myeloma. Traditionally, clonality is established using DNA by the demonstration of the presence of a uniquely rearranged V(D)J clone. It is accepted that cancer arises in one lymphoid cell; after rearrangement and in the uncontrolled proliferation of this cell, the clonotype is largely preserved. Although somatic hypermutation may occur, the V gene and CDR3 nucleotide sequences are maintained and the clonotype does not change. Therefore, cancer can be monitored by testing for the presence of this rearranged clonotype. In general, monitoring takes place by defining the T‐cell or B‐cell clonotype that was detected in the diagnostic lymphoma, leukemia, or multiple myeloma sample; then this clone is followed in subsequent analysis (clonotype‐informed).

The rearranged clone is commonly analyzed and quantified by PCR amplification or NGS of the DNA of the CDR3 segment. When NGS is used, the Ig/TCR gene family is defined from the DNA sequence; then, this family is followed and monitored as a part of MRD monitoring.

In this paper, we are addressing the potential of using peripheral blood cfRNA for the establishment of clonality in lymphoid neoplasms and for potential monitoring. While every cell contains one copy of the expressed rearranged Ig or TCR, numerous copies of the expressed RNA are typically present in each cell. The abundance of Ig/TCR RNA in lymphoid cells may increase the sensitivity of detecting and monitoring clonality [13]. Current advances in NGS allow us to quantify mRNA and determine the sequence and the expressed family very reliably and reproducibly. Furthermore, for NGS in routine clinical testing, sequencing the Ig/TCR intronless RNA is significantly more cost‐effective and practical than sequencing the entire genomic loci of Ig/TCR with all their introns.

We have previously reported our approach to isolate and sequence cfRNA in reliable and reproducible fashion [14, 15]. Our approach for determining clonality by cfRNA is to sequence the entire Ig and TCR RNA by hybrid capture and NGS, then define the top expressed gene families (clonotypes) and compare their relative expression levels to determine the presence or absence of clonality. To establish the expression level of a clonotype as compared to other clones, we tested a large number of tissue samples known to be either positive or negative for B‐ or T‐cell neoplasms as confirmed by either flow cytometry/immunohistochemistry (IHC) or PCR assays. Using a ratio of the highest expressed clone or gene family to the second highest clone of 10 was a stringent enough cutoff point to classify 100% of known negative control samples as negative. We excluded the TRA expression profile in this validation (see below). However, this stringent criterion misclassified 6% of the clonal cases as negative (100% specificity and 94% sensitivity), which is acceptable for clonotype naive testing. In clinical practice, clearly a ratio between 5 and 10 should be considered in the context of other findings, such as mutations, flow cytometry/IHC, or morphology for determining clonality.

After establishing the cutoff point for clonality, we analyzed cfRNA data in a large number of plasma samples from patients known to have hematologic and solid tumors as well as samples from normal individuals or individuals with CHIP. These peripheral blood samples showed evidence of circulating tumor DNA and RNA with various levels of disease. Majority of these samples were tested to evaluate MRD based on mutation profiling.

For the demonstration of Ig clonality, we analyzed clonality in the heavy chain (IgH) and light chains kappa (IgK) and lambda (IgL). As shown in Table 2, only 7% of all normal individuals or individuals with CHIP showed evidence of clonality for B‐cell in at least one of these markers. Most of the tested samples were from individuals at advanced age and this prevalence is expected to be high in this age group based on the reported prevalence of monoclonal B‐cell lymphocytosis (MBL) [16]. The median age of this tested group was 66 years. Similarly, T‐cell clonality was detected using either TRB or TRG in 3% of normal/CHIP population. This also has been reported in normal individuals as T‐cell clones of uncertain significance (T‐CUS) [17, 18].

We were able to demonstrate clonality in 36% of cfRNA of patients with B‐cell neoplasms or multiple myeloma. In T‐cell neoplasms, we detected clonality meeting the 10‐fold cutoff in 22% of cases. Using TRA after excluding TRAV1‐1 gene family increased the detection rate by 2% (total 25%). TRA is known to have limited diversity and TRAV1‐1 appears to be dominantly expressed, which reduces the specificity if used for determining clonality. To increase specificity, using two rearranged receptors should always be attempted whenever possible. This testing is based on detecting 10‐fold clonal dominance as compared with other clonotypes. For monitoring when the clonotype is predefined based on testing a diagnostic neoplastic sample (clonotype‐informed), 10‐fold dominances are not required and the detection of the same clone is adequate for confirming MRD. Therefore, the sensitivity of this approach is expected to be significantly higher if testing is clonotype‐informed. Furthermore, the majority of the cases included in this testing contained very little disease in circulation. For screening, this data suggests that clonality alone is inadequate for diagnosis and mutation profiling as well as chromosomal abnormalities (CNV) should also be considered. In addition, 3% of the lymphoid group showed clonality for both T cells and B cells, which also suggests that mutation profiling is necessary for proper interpretation of findings.

B‐ and T‐cell clonality was detected in patients with myeloid neoplasms and solid tumors (Table 2). While cross‐lineage rearrangements/lineage infidelity has been described [8], this could also be due to either the co‐presence of lymphoid neoplasms or due to a reactive process in response to the solid or myeloid neoplastic process. Also, we cannot rule out the possibility that the clonality in circulation reflects clonal proliferation of lymphoid cells at the tumor site. The data suggest that while clonality alone should not be used to determine the presence or absence of lymphoid neoplasm, it might provide information that can be useful to evaluate the overall health of the immune system.

This study is limited by not comparing the findings in the peripheral blood cfRNA with the findings in simultaneously collected tissue samples. While this head‐to‐head comparison can be performed in cases with localized tumors, it is difficult to perform when the tumor is at a low level. Furthermore, the current study does not address the potential of false negative cases due to somatic hypermutations and misclassification of expression gene families. At any rate, the current study is the first of its kind showing that using cfRNA is a practical and reliable approach to detect B‐ and T‐cell clonality. Further studies are needed to determine the practicality and sensitivity of this approach for detecting and monitoring MRD, especially when combined with mutation profiling.

## Conflict of interest

AA, AC, SA, and MA work for a diagnostic company offering clinical clonality testing.

## Author contributions

MA, AA, AG, and DS contributed to the conceptualization. MA, AA, SA, and AC contributed to the methodology and data curation. MA, AA, SA, AC, AG, and DS contributed to the analysis. MA, AA, SA, AC, AG, DS, PP, DV, HP, AP, and AI contributed to the investigation, resources, and writing—review and editing. All authors have read and agreed to the published version of the manuscript.

## Data Availability

Raw data are available upon request to the first author.

## References

[1] van den Brand M , Möbs M , Otto F , Kroeze LI , de Castro DG , Stamatopoulos K , Davi F , Bravetti C , Kolijn PM , Vlachonikola E *et al*. (2023) EuroClonality‐NGS recommendations for evaluation of B‐cell clonality analysis by next‐generation sequencing: a structured approach with the DEPART algorithm. J Mol Diagn 25, 729–739. doi: 10.1016/j.jmoldx.2023.06.011 37467928

[2] Short NJ , Jabbour E , Albitar M , de Lima M , Gore L , Jorgensen J , Logan AC , Park J , Ravandi F , Shah B *et al*. (2019) Recommendations for the assessment and management of measurable residual disease in adults with acute lymphoblastic leukemia: a consensus of north American experts. Am J Hematol 94, 257–265. doi: 10.1002/ajh.25338 30394566 PMC6572728

[3] Stewart JP , Gazdova J , Darzentas N , Wren D , Proszek P , Fazio G , Songia S , Alcoceba M , Sarasquete ME , Villarese P *et al*. (2021) Validation of the EuroClonality‐NGS DNA capture panel as an integrated genomic tool for lymphoproliferative disorders. Blood Adv 5, 3188–3198. doi: 10.1182/bloodadvances.2020004056 34424321 PMC8405189

[4] van Dongen JJM , Langerak AW , Brüggemann M , Evans P a S , Hummel M , Lavender FL , Delabesse E , Davi F , Schuuring E , García‐Sanz R *et al*. (2003) Design and standardization of PCR primers and protocols for detection of clonal immunoglobulin and T‐cell receptor gene recombinations in suspect lymphoproliferations: report of the BIOMED‐2 concerted action BMH4‐CT98‐3936. Leukemia 17, 2257–2317. doi: 10.1038/sj.leu.2403202 14671650

[5] Monter A and Nomdedéu JF (2019) ClonoSEQ assay for the detection of lymphoid malignancies. Expert Rev Mol Diagn 19, 571–578. doi: 10.1080/14737159.2019.1627877 31179776

[6] Cavagna R , Guinea Montalvo ML , Tosi M , Paris M , Pavoni C , Intermesoli T , Bassan R , Mosca A , Rambaldi A and Spinelli O (2020) Capture‐based next‐generation sequencing improves the identification of immunoglobulin/T‐cell receptor clonal markers and gene mutations in adult acute lymphoblastic leukemia patients lacking molecular probes. Cancers 12, 6. doi: 10.3390/cancers12061505 PMC7352935 32526928

[7] Singh RR (2022) Target enrichment approaches for next‐generation sequencing applications in oncology. Diagnostics 12, 1539. doi: 10.3390/diagnostics12071539 35885445 PMC9318977

[8] Wang H‐W and Raffeld M (2019) Molecular assessment of clonality in lymphoid neoplasms. Semin Hematol 56, 37–45. doi: 10.1053/j.seminhematol.2018.05.008 30573043

[9] Rossi D , Spina V , Bruscaggin A and Gaidano G (2019) Liquid biopsy in lymphoma. Haematologica 104, 648–652. doi: 10.3324/haematol.2018.206177 30846503 PMC6442975

[10] Roschewski M , Dunleavy K , Pittaluga S , Moorhead M , Pepin F , Kong K , Shovlin M , Jaffe ES , Staudt LM , Lai C *et al*. (2015) Comparative study of circulating tumor DNA and computerized tomography monitoring in untreated diffuse large B‐cell lymphoma. Lancet Oncol 16, 541–549. doi: 10.1016/S1470-2045(15)70106-3 25842160 PMC4460610

[11] Kurtz DM , Green MR , Bratman SV , Scherer F , Liu CL , Kunder CA , Takahashi K , Glover C , Keane C , Kihira S *et al*. (2015) Noninvasive monitoring of diffuse large B‐cell lymphoma by immunoglobulin high‐throughput sequencing. Blood 125, 3679–3687. doi: 10.1182/blood-2015-03-635169 25887775 PMC4463733

[12] Gong Q , Wang C , Zhang W , Iqbal J , Hu Y , Greiner TC , Cornish A , Kim J‐H , Rabadan R , Abate F *et al*. (2017) Assessment of T‐cell receptor repertoire and clonal expansion in peripheral T‐cell lymphoma using RNA‐seq data. Sci Rep 7, 11301. doi: 10.1038/s41598-017-11310-0 28900149 PMC5595876

[13] Bashford‐Rogers RJ , Palser AL , Idris SF , Carter L , Epstein M , Callard RE , Douek DC , Vassiliou GS , Follows GA , Hubank M *et al*. (2014) Capturing needles in haystacks: a comparison of B‐cell receptor sequencing methods. BMC Immunol 15, 29. doi: 10.1186/s12865-014-0029-0 25189176 PMC4243823

[14] Albitar M , Zhang H , Charifa A , Ip A , Ma W , McCloskey J , Donato M , Siegel D , Waintraub S , Gutierrez M *et al*. (2023) Combining cell‐free RNA with cell‐free DNA in liquid biopsy for hematologic and solid tumors. Heliyon 9, e16261. doi: 10.1016/j.heliyon.2023.e16261 37251903 PMC10208940

[15] Albitar M , Charifa A , Agersborg S , Pecora A , Ip A and Goy A (2024) Expanding the clinical utility of liquid biopsy by using liquid transcriptome and artificial intelligence. J Liq Biopsy 6, 100270. doi: 10.1016/j.jlb.2024.100270 40027317 PMC11863701

[16] Semenzato G , Teramo A , Calabretto G and Zambello R (2024) T‐cell clones of uncertain significance. When is the rogue clone dangerous? Haematologica 110, 37–46. doi: 10.3324/haematol.2024.286023 PMC11694120 39363880

[17] El‐Sharkawi D and Dearden C (2025) Diagnosis and Management of Large Granular Lymphocytic Leukaemia and its Differentiation from T Cell Clones of uncertain significance. Hematol Oncol 43, e70076. doi: 10.1002/hon.70076 40517544

[18] Shi M , Olteanu H , Jevremovic D , He R , Viswanatha D , Corley H and Horna P (2020) T‐cell clones of uncertain significance are highly prevalent and show close resemblance to T‐cell large granular lymphocytic leukemia. Implications for laboratory diagnostics. Mod Pathol 33, 2046–2057. doi: 10.1038/s41379-020-0568-2 32404954

